# Antioxidant potential of theaflavin ameliorates the activities of key enzymes of glucose metabolism in high fat diet and streptozotocin – induced diabetic rats

**DOI:** 10.1080/13510002.2019.1624085

**Published:** 2019-05-29

**Authors:** Kirubananthan Gothandam, Vijayan Siva Ganesan, Thangaraj Ayyasamy, Sundaram Ramalingam

**Affiliations:** a Department of Biotechnology, University of Madras, Chennai, India; b Department of Plant Biology and Plant Biotechnology, Government Arts College for Men (Autonomous), Nandanam, University of Madras, Chennai, India; c Department of Medical Biochemistry, Dr. ALM Post Graduate Institute of Basic Medical Sciences, University of Madras, Chennai, India; d Department of Biochemistry, Saveetha Dental College & Hospital, Saveetha Institute of Medical & Technical Sciences, Chennai, India

**Keywords:** Theaflavin, diabetes, metabolic enzymes, glycogen, antioxidant enzymes

## Abstract

**Objectives:** The present study was to evaluate the effect of theaflavin on the activities of key enzymes of carbohydrate metabolism in high fat diet and streptozotocin – induced diabetic rats.

**Methods:** Diabetes was induced in male albino Wistar rats by feeding them with high fat diet comprising of standard laboratory rat chow 84.3%, lard 5%, egg yolk powder 10%, cholesterol 0.2% and bile salt 0.5% for 2 weeks. After 2 weeks, the animals were kept in an overnight fast and injected with low dose of streptozotocin (40 mg/kg b.w).

**Results:** Theaflavin (100 mg/kg b.w /day) was administered orally to diabetic rats for 30 days. At the end of the experimental period, diabetic control rats showed significant increase in plasma glucose, homeostatic model assessment of insulin resistance (HOMA-IR), glycosylated hemoglobin (HbA1c) with concomitant decrease in plasma insulin, total hemoglobin and body weight. The activities of key enzymes of carbohydrate metabolism, lipid peroxidation markers, antioxidant enzymes, glycogen content and glycogen synthase and glycogen phosphorylase were also altered in diabetic rats.

**Discussion:** Oral administration of theaflavin to diabetic rats significantly ameliorated all the biochemical alterations to near normal levels. The results of the present study suggest that theaflavin exhibits antidiabetic effect through its antioxidant activity.

## Introduction

Diabetes mellitus is a metabolic disorder which is characterized by hyperglycemia **along with** alterations in carbohydrate, lipid and protein metabolism associated with complete or relative deficiencies in insulin secretion and/or insulin action [1]. Chronic hyperglycemia in diabetes is associated with long term damage, dysfunction and eventually the failure of organs, particularly the eyes, kidneys, nerves and cardiovascular system [2]. In addition to hyperglycemia, several other factors such as dyslipidemia or hyperlipidemia are also involved in the development of cardiovascular complications in diabetes which are the major causes of morbidity and mortality [3]. The World Health Organization (WHO) estimates that more than 220 million people worldwide have diabetes and this number would be likely to more than double by the year 2030 [4]. The management of diabetes with insulin and synthetic oral hypoglycemic drugs can produce serious side effects and in addition, they fail to prevent diabetes related complications in many patients. Therefore, new diabetic management strategy is obligatory nowadays which should be more effective and have less side effects than the synthetic drugs [5]. Many species of plants and plant based drugs have been described in the scientific literature as having hypoglycemic activity due to their apparent effectiveness and minimal side effects in clinical experience and relatively low costs [6].

**Figure 1.**
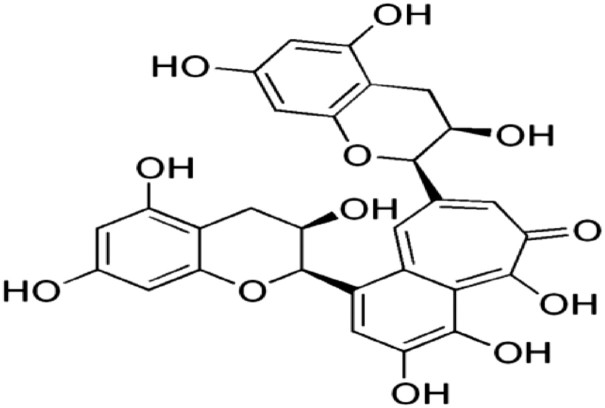
Structure of theaflavin.

Theaflavins are major chemical constituents of black tea which have been reported to have antioxidant [7], anti-cancer [8], antiinflammatory [9], antimicrobial [10] and antiviral abilities, including bovine coronavirus, bovine rotavirus [11], HIV-1 [12,13], influenza and HSV-1 [14]. Etc. However, the antioxidant potential of theaflavin to ameliorate the activities of key enzymes of glucose metabolism in high fat diet and streptozotocin – induced diabetic rats has not been explored so far. Therefore, the present study was aimed to evaluate effect of theaflavin on glucose metabolizing enzymes in diabetic rats. The structure of theaflavin was given Figure 1.

## Materials and methods

### Chemicals and drugs

Theaflavin, streptozotocin and high fat diet components such as cholesterol, bile salt, egg yolk power and lard were obtained from Sigma Chemical Company (St. Louis, MO, USA), Sisco Research Laboratories Pvt. Ltd., Mumbai, India, Central Drug House Pvt. Ltd., New Delhi, India, SKM Egg Products Export (India) Limited, Erode, Tamil Nadu, India and lard was obtained from local market in Chennai. All other chemicals used were of analytical grade.

### Animal

Male albino Wistar rats weighing 200–220 g body weight were procured from the Central Animal House Facility, University of Madras, Taramani Campus, Chennai, Tamil Nadu, India. They were maintained at an ambient temperature of 25 ± 2°C and 12/12 h of light/dark cycle. Animals were given standard commercial rat chow and water ad libitum and housed under standard environmental conditions throughout the study. The experiments were conducted according to the ethical norms approved by the Ministry of Social Justices and Empowerment, Government of India and Institutional Animal Ethics Committee Guidelines (IAEC No 32/02/2014).

### Induction of type 2 diabetes in rats

The animals were divided into seven groups of six animals each. The rats were fed with high fat diet consisting of 84.3% (w/w), standard laboratory chow, 5% lard (v/v) 10% yolk powder (w/w), cholesterol 0.2% (w/w) and 0.5% (w/w) bile salt for 2 weeks [15]. After 2 weeks, the animals were kept in an overnight fast and injected with low dose of streptozotocin (40 mg/kg, dissolved in 0.1 M sodium citrate buffer, pH 4.5) [16]. Fasting blood glucose was measured three days after the injection. The rats with fasting blood glucose levels above 250 mg/dL were considered diabetic. The diabetic rats were fed on the high-fat diet for another 4 weeks.

## Experimental design

In this experiment, a total of 42 rats (30 diabetic surviving rats 12 normal rats) were divided into six groups of six rats in each.

**Table:** 

Group I:	Normal control (received 0.5 mL of distilled water).
Group II:	Drug control (normal healthy control rats received intra gastrically theaflavin (100 mg/kg b.wt.) dissolved in 0.5 mL of distilled water for 30 days.
Group III:	Diabetic control.
Group IV:	Diabetic rats received intra gastrically theaflavin (25 mg/kg b.wt.) dissolved in 0.5 mL of distilled water for 30 days.
Group V:	Diabetic rats received intra gastrically theaflavin and metformin (50 mg/kg b.wt.) dissolved in 0.5 mL of distilled water for 30 days.
Group VI:	Diabetic rats received intra gastrically theaflavin (100 mg/kg b.wt.) dissolved in 0.5 mL of distilled water for 30 days.
Group VII:	Diabetic rats received intra gastrically Metformin (500 mg/kg b.wt.) dissolved in 0.5 mL of distilled water for 30 days.

## Sample collection

After 30 days of treatment, the animals were deprived of food overnight and sacrificed by decapitation. Blood was collected in two different tubes, i.e. one with a mixture of potassium oxalate and sodium fluoride (1:3) for estimation of plasma insulin and glucose and another with ethylene diamine tetra acetic acid (EDTA) for the estimation of hemoglobin and glycated hemoglobin. Liver and kidney tissues were excised immediately and rinsed in ice-chilled normal saline to remove the blood. Known weights of the tissues were minced and homogenized in 5.0 ml of 0.1M Tris–HCl buffer (pH 7.4) in ice cold condition. The homogenate was centrifuged and the supernatant was used for the estimation of various biochemical parameters. A section of pancreas was kept aside for histological studies. Body weights of all the animals were recorded prior to the treatment and sacrifice. Food and water intake of all groups of animals were monitored on a daily basis for 30 days at a fixed time. Fixed amount of rat chow and fluid was given to each rats and replenished the next day.

## Biochemical analysis

Plasma glucose was estimated by the method of Trinder using a reagent kit Trinder [17], Haemoglobin (Hb) and glycated haemoglobin (HbA1c) were estimated by the method of Drabkin and Austin [18] and Sudhakar and Pattabiraman [19] respectively. The plasma insulin was measured by the method of Burgi et al. [20]. Carbohydrate metabolizing enzymes such as hexokinase, pyruvate kinase, lactate dehydrogenase, glucose-6-phosphatase, fructose-1,6-bisphosphatase, glucose-6-phosphate dehydrogenase, glycogen synthase, glycogen phosphorylase and glycogen content were estimated by the method of Brandstrup et al. [21], Pogson and Denton [22], King [23], Koide and Oda [24], Gancedo and Gancedo [25], Ells and Kirkman [26], Leloir and Goldemberg [27], Cornblath et al. [28] and Morales et al. [29] respectively. Lipid peroxidation and hydroperoxides were estimated in liver and kidney tissues by the method of Niehius and Samuelsson [30] and Jiang et al. [31], respectively. Catalase (CAT) superoxide dismutase (SOD), glutathione peroxidase (GPx), glutathione-S-transferase (GST), ascorbic acid (vitamin C), α -tocopherol (Vitamin E), GSH and protein were determined by the method of Sinha [32]. Stringer et al. [33], Rotruck et al. [34] Habig et al. [35] Omaye et al. [36] Baker et al. [37], Ellman [38] and Lowry et al. [39] respectively.

HOMA−IR=fastinginsulin×fastingbloodsugar/405

## Estimation of oral glucose tolerance test (OGTT)

Oral glucose tolerance test (OGTT) was performed according to the method of Du Vigneaud and Karr [40]. After overnight fasting, ‘0’ minute blood samples (0.2 mL) were taken from control and experimental rats. Without delay, a glucose solution (2 g/kg body weight) was administered by oral gavage. Blood samples were taken at 30, 60, 90 and 120 min after glucose administration. Blood samples were collected with potassium oxalate and sodium fluoride and glucose levels were determined by the kit method of Trinder [15].

## Histopathology of pancreas

The pancreatic tissues of the tested rats were fixed in 10% V/V formaldehyde, dried out in an evaluated arrangement of ethanol and embedded in paraffin. Pancreatic sections (5 μm thick) were acquired utilizing rotary microtome and afterward rehydrated. Sections were then stained by hematoxylin–eosin (H&E) and viewed under the light microscope to assess the presence of β cells in the pancreatic tissue of various group. Digital images were obtained using an Olympus BX51 microscope equipped with a Camedia C3040ZOOM digital camera (Olympus America Inc., Melville, NY, USA). All images were taken under 40x magnifications.

## Statistical analysis

The results are expressed as mean ± SD. Differences between groups were assessed by ANOVA using the SPSS/17 student software package for windows. Post hoc testing was performed for inter-group comparisons using the least significance difference (LSD) *P*-values < 0.05 were considered as significantly altered.

## Results

### Dose dependent effects of theaflavin on plasma glucose, insulin levels and HOMA-IR index

Blood glucose, plasma insulin levels and homeostatic model assessment of insulin resistant of normal and experimental rats are given in Table 1. The diabetic rats showed a significant increase in blood glucose and a significant decrease in plasma insulin levels. Administration of theaflavin (25, 50 and 100 mg/kg b.w.) at different doses to diabetic rats caused a significant decrease in blood glucose levels and a significant increase in plasma insulin when compared with diabetic untreated rats. In the same context, diabetic rats showed a significant increase in HOMA-IR when compared with the control rats. Supplementation of different concentrations (25, 50 and100 mg/kg b.w.) of theaflavin significantly decreased the HOMA-IR index in a dose dependent manner. But, significantly more pronounced effect of theaflavin was observed at a dose of 100 mg/kg body weight when compared with other two doses of the drug and the effect of the drug was comparable to that of metformin. Therefore, 100 mg/kg body weight was fixed as an effective dose and used for further analysis.

**Table 1.** Dose dependent effect of theaflavin on blood glucose, insulin levels and HOMA-IR index in control and experimental animals.

Parameters	Control	Normal + Theaflavin (100 mg/kgb.wt)	Diabetes	Diabetes + Theaflavin (25 mg/kg b.wt)	Diabetes + Theaflavin (50 mg/kg b.wt)	Diabetes + Theaflavin 100 mg/kgb.wt	Diabetes + Metformin (500 mg/kg b.wt)
Glucose (mg/dl)	97.65 ± 5.03	95.28 ± 5.15	273.76 ± 16.35^b^	239.43 ± 12.54^c^	183.98 ± 8.74^d^	131.81 ± 7.49^e^	129.64 ± 8.12
Insulin (µU/ml)	19.42 ± 1.80	18.99 ± 1.82	9.57 ± 1,18^b^	11.80 ± 1.12^c^	13.51 ± 1.36^d^	16.15 ± 1.35^e^	17.66 ± 1.27
HOMA-IR	4.68 ± 0.64	4.38 ± 0.53	6.62 ± 0.70^b^	7.20 ± 0.81^c^	6.2 ± 0.58^d^	5.13 ± 0.37^e^	5.57 ± 0.49

Notes: Values are given as mean ± SD for six animals in each group. Values are considered significantly different at *P* < 0.05 with post hoc LSD test *P < 0.05.

^a^Control vs. Drug control (Theaflavin alone treated rats).

^b^Control rats vs. Diabetic rats.

^c^Diabetic rats vs. Theaflavin 25 mg/kg.

^d^Diabetic rats vs. Theaflavin 50 mg/kg.

^e^Diabetic rats vs. Theaflavin 100 mg/kg.

^f^Diabetic rats treated with Theaflavin 100 mg/kg vs. Diabetic rats treated with metformin (500 mg/kg).

### Effect of theaflavin on oral glucose tolerance test (OGTT)

**Figure 2.**
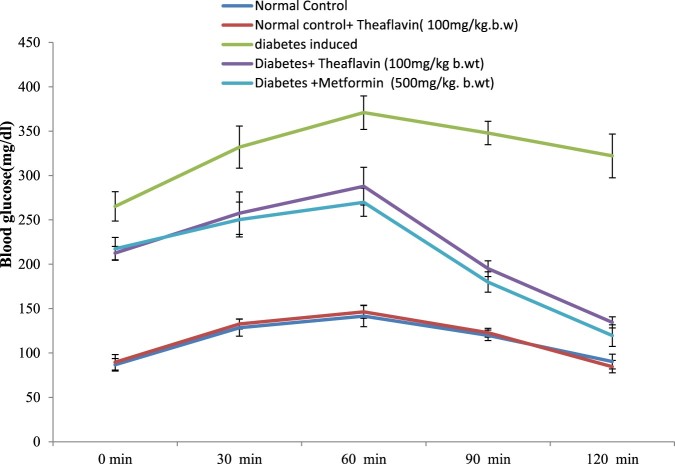
Effect of theaflavin on oral glucose tolerance test. Each value is mean ± S.D. for 6 rats (*n* = 6) in each group.

Results of OGTT conducted on control and different experimental groups are shown in Figure 2. After the oral administration of glucose in normal control rats, the blood glucose levels reached the fasting levels at 2 h whereas in diabetic control rats, the blood glucose levels remained at peak even after 2 h. Theaflavin supplementation prevented the rise of blood glucose levels significantly in a dose dependent manner. However, the prominent glucose lowering effect was found at a dose of 100 mg/kg body weight when compared to other two doses (25 and 50 mg/kg b. w.) at 60 min. The results were almost similar to that of diabetic rats treated with metformin when compared to untreated diabetic rats. No significant changes were observed in the rats administered with theaflavin alone when compared with control rats.

### Effect of theaflavin on the changes in the body weight, food and water intake

The changes in the body weight, food and water intake in control and experimental rats were depicted in Table 2. Food and water intake were elevated whereas the body weight significantly decreased in diabetic rats compared with normal control rats. The daily administration of theaflavin and metformin to diabetic rats for 30 days caused a significant reduction in food and fluid intakes and an increase in the body weight when compared with diabetic control rats.

**Table 2.** Effect of theaflavin on body weight, fluid intake and food intake of control and experimental animals.

Groups	Body weight(g)		Food intake (g/rat/day)		Water intake (ml/rat/day)	
	Initial	Final	Before	After	Before	After
Control	210.67 ± 6.89	239.67 ± 10.27	15.54 ± 1.21	14.14 ± 1.31	75.58 ± 5.57	73.64 ± 4.36
Normal + Theaflavin (100 mg/kg b.wt)	214.00 ± 7.69	242.83 ± 9.11	16.38 ± 1.19	14.05 ± 1.34	76.52 ± 6.11	78.03 ± 5.47
Diabetes induced	209.17 ± 8.11	150.83 ± 9.45^b^	58.50 ± 7.21	78.01 ± 6.72^b^	145.11 ± 10.55	155.33 ± 11.84^b^
Diabetic + Theaflavin (100 mg/kg b.wt)	208.98 ± 7.12	196.67 ± 9.27^c^	42.03 ± 4.19	30.85 ± 2.16^c^	135.41 ± 5.27	78.05 ± 6.10^c^
Diabetic + Metformin (500 mg/kg b.wt)	207.67 ± 6.86	199.83 ± 7.13	43.77 ± 2.94	29.04 ± 1.88^c^	136.12 ± 4.96	76.87 ± 6.37

Notes: Values are given as mean ± SD for six animals in each group. Values are considered significantly different at *P* < 0.05 with post hoc LSD test *P < 0.05.

^a^Control vs. Drug control (Theaflavin alone treated rats).

^b^Control rats vs. Diabetic rats.

^c^Diabetic rats vs. Theaflavin 100 mg/kg.

^d^Diabetic rats treated with Theaflavin 100 mg/kg vs. Metformin (500 mg/kg).

### Effect of theaflavin on the levels of hemoglobin and glycosylated hemoglobin

The levels of hemoglobin and HbA1c in control and experimental animals were depicted in Table 3. The diabetic rats showed significant decrease in the level of total hemoglobin and significant increase in the levels of HbA1C when compared to control rats. The levels of total hemoglobin and HbA1C were significantly reversed by the administration of theaflavin and metformin to diabetic rats. Normal rats treated with theaflavin at a dose of 100 mg/kg body weight did not show any significant changes in plasma glucose, insulin, and hemoglobin and HbA1C levels.

**Table 3.** Effects of theaflavin on hemoglobin and glycosylated hemoglobin levels in control and experimental animals.

Parameters	Control	Normal+ Theaflavin (100 mg/kg b.wt)	Diabetes	Diabetes + Theaflavin (100 mg/kg b.wt)	Diabetes + Metformin
Haemoglobin(g/dl)	14.62 ± 1.15	13.93 ± 0.87	7.38 ± 1.33^b^	11.96 ± 1.30^c^	12.87 ± 1.11
HbA1c (%)	4.75 ± 0.41	4.64 ± 0.42	11.15 ± 1.16^b^	7.28 ± 0.73^c^	6.40 ± 0.78

Notes: Values are given as mean ± SD for six animals in each group. Values are considered significantly different at *P* < 0.05 with post hoc LSD test *P < 0.05.

^a^Control vs. Drug control (Theaflavin alone treated rats).

^b^Control rats vs. Diabetic rats.

^c^Diabetic rats vs. Theaflavin 100 mg/kg.

^d^Diabetic rats treated with Theaflavin 100 mg/kg vs. Metformin (500 mg/kg).

### Effects of theaflavin on liver and muscle glycogen content and the levels of glycogen synthase and glycogen phosphorylase in liver

A significant decrease in muscle and liver glycogen content as well as in the glycogen synthase activity and a concomitant increase in the activity of glycogen phosphorylase were observed in the liver of diabetic rats when compared to normal rats. Oral administration of theaflavin and metformin to diabetic rats reinstated the glycogen content and the activities of glycogen synthase and glycogen phosphorylase to near normal levels in the respective tissues when compared to untreated diabetic rats. Normal rats treated with theaflavin did not show any significant changes in the glycogen content as well as in the glycogen synthase and glycogen phosphorylase (Table 4).

**Table 4.** Effects of theaflavin on glycogen metabolism in control and experimental animals.

Groups	Glycogen synthase (*µ* moles of UDP formed/h/mg protein)	Glycogen phosphorylase (*µ* moles Pi liberated/h/mg protein)	Liver glycogen (mg/g tissue)	Muscle glycogen (mg/g tissue)
Control	820.16 ± 7.13	587.33 ± 7.20	46.83 ± 2.48	17.50 ± 1.64
Normal + Theaflavin 100 mg/kg b.wt	817.66 ± 6.77	586.50 ± 5.20	47.66 ± 2.58	17.83 ± 1.47
Diabetes	514.83 ± 13.22	787.50 ± 11.58^b^	21.83 ± 4.40^c^	10.66 ± 0.81
Diabetic + Theaflavin 100 mg/kg b.wt	795.66 ± 10.63	660.83 ± 9.17^b^	35.50 ± 4.37^c^	15.50 ± 1.51
Diabetic + Metformin 100 mg/kg b.wt	798.40 ± 11.20	662.55 ± 8.46	37.67 ± 4.80	17.80 ± 1.42

Notes: Values are given as mean ± SD for six animals in each group. Values are considered significantly different at *P* < 0.05 with post hoc LSD test *P < 0.05.

^a^Control vs. Drug control (Theaflavin alone treated rats).

^b^Control rats vs. Diabetic rats.

^c^Diabetic rats vs. Theaflavin 100 mg/kg.

^d^Diabetic rats treated with Theaflavin 100 mg/kg vs. Metformin (500 mg/kg).

### Effects of theaflavin on glucose metabolism

The effect of theaflavin supplementation on the activities of hexokinase, pyruvate kinase, lactate dehydrogenase, glucose-6-phosphatase, fructose-1,6- bisphosphatase and glucose-6-phosphate dehydrogenase in liver and kidney tissues of control and experimental rats are shown in Table 5. There were no significant changes in the levels of these parameters in control rats treated with theaflavin alone. However, the activities of hexokinase, pyruvate kinase and glucose-6-phosphate dehydrogenase were significantly decreased whereas the activities of glucose-6-phosphatase and fructose-1,6-bisphosphatase and lactate dehydrogenase were significantly increased in liver and kidney tissues of diabetic rats. Treatment with theaflavin and metformin to diabetic rats, the altered activities of these enzymes were reinstated to near normalcy in liver and kidney tissues.

**Table 5.** Effects of theaflavin on carbohydrate metabolic enzymes in control and experimental.

Parameters	Control	Normal + Theaflavin (100 mg/kg b.wt)	Diabetes	Diabetes + Theaflavin (100 mg/kg b.wt)	Diabetes + Metformin (500 mg/kg b.wt)
Liver					
Hexokinase	252.35 ± 14.90	250.97 ± 14.74	153.93 ± 9.65^b^	226.04 ± 14.17^c^	225.13 ± 13.59
pyruvatekinase	200.96 ± 15.66	202.16 ± 15.7	104.47 ± 10.19^b^	181.10 ± 12.51^c^	179.24 ± 12.6
LDH	232.28 ± 12.15	230.05 ± 11.74	414.31 ± 23.50^b^	249.74 ± 11.42^c^	247.57 ± 11.78
Glucose 6 Phosphatase	730.08 ± 47.29	728.00 ± 47.57	1558.39 ± 55.09^b^	766.78 ± 49.47^c^	765.30 ± 49.42
Fructose 1,6 bisphosphatase	470.65 ± 23.35	467.99 ± 23.41	804.44 ± 55.18^b^	500.76 ± 27.29^c^	498.82 ± 27.17
glucose 6 phosphate dehydrogenase	510.47 ± 39.41	507.45 ± 38.96	239.24 ± 21.25^b^	489.58 ± 35.89^c^	487.45 ± 35.90
Kidney					
Hexokinase	152.35 ± 14.99	149.56 ± 14.97	82.17 ± 5.59^b^	130.87 ± 13.84^c^	129.65 ± 13.58
Pyruvatekinase	130.55 ± 11.48	128.12 ± 11.74	70.38 ± 9.57^b^	100.02 ± 10.56^c^	98.71 ± 10.51
LDH	454.12 ± 24.15	451.09 ± 24.01	750.41 ± 49.73^b^	486.35 ± 29.01^c^	484.71 ± 29.08
Glucose Phosphatase	381.33 ± 25.63	378.85 ± 25.75	751.33 ± 32.08^b^	423.98 ± 29.45^c^	422.11 ± 29.53
Fructose 1,6 bisphosphatase	714.58 ± 52.01	711.95 ± 52.19	912.74 ± 81.91^b^	794.86 ± 55.40^c^	793.15 ± 55.50
glucose 6 phosphate dehydrogenase	657.77 ± 44.71	651.2 ± 40.70	276.50 ± 23.59^b^	601.44 ± 40.67^c^	600.08 ± 41.18

Notes: Values are given as mean ± SD for six animals in each group. Values are considered significantly different at *P* < 0.05 with post hoc LSD test *P < 0.05. Units are expressed as: µmoles of glucose-6-phosphate formed/h/mg of protein for hexokinase, µmoles of pyruvate formed/min/mg of protein for pyruvate kinase, µmoles of pyruvate formed/h/mg of protein for lactate dehydrogenase, µmoles of Pi liberated/h/mg of protein for glucose-6- phosphatase and fructose-1, 6-bisphosphatase and µmoles of NADPH/min/mg of protein for glucose-6-phosphate dehydrogenase.

^a^Control vs. Drug control (Theaflavin alone treated rats).

^b^Control rats vs. Diabetic rats.

^c^Diabetic rats vs. Theaflavin 100 mg/kg.

^d^Diabetic rats treated with Theaflavin 100 mg/kg vs. Metformin (500 mg/kg).

### Estimation of lipid peroxidation markers

Table 6 shows the levels of TBARS and Hydroperoxides in liver and kidney tissues of control and experimental rats. Diabetic rats showed increased levels of TBARS and Hydroperoxides when compared to normal control rats. Oral administration of theaflavin to diabetic rats significantly decreased lipid peroxidation markers in liver and kidney tissues.

**Table 6.** Effect of Theaflavin on the levels of TBARS and HP in the tissues of control and experimental rats.

Groups	TBARS (mM/100 g tissue)		Hydroperoxides (mM/100 g tissue)	
	Liver	Kidney	Liver	Kidney
control	0.83 ± 0.05	1.32 ± 0.09	72.03 ± 5.11	41.85 ± 3.55
Normal + Theaflavin (100 mg/kg b.wt)	0.86 ± 0.07	1.26 ± 0.08	70.25 ± 4.95	39.09 ± 3.73
Diabetes Induced	1.88 ± 0.15^b^	2.45 ± 0.23^b^	137.25 ± 9.95^b^	76.73 ± 7.47^b^
Diabetic + Theaflavin (100 mg/kg b.wt)	1.19 ± 0.08^c^	1.40 ± 0.12^c^	97.21 ± 8.21^c^	52.04 ± 4.65^c^
Diabetic + Metformin (500 mg/kg b.wt)	1.01 ± 0.09	1.20 ± 0.10	92.51 ± 8.28	48.56 ± 4.38

Notes: Values are given as mean ± SD for six animals in each group. Values are considered significantly different at *P* < 0.05 with post hoc LSD test *P < 0.05.

^a^Control vs. Drug control(Theaflavin alone treated control rats).

^b^Control rats vs. Diabetic rats.

^c^Diabetic rats vs. Theaflavin treated diabetic rats.

^d^Theaflavin treated diabetic rats vs. Metformin treated diabetic rats.

### Estimation of enzymatic and non enzymatic antioxidant enzymes

Tables 7 and 8 illustrate the activities of enzymatic antioxidants (SOD, CAT, GPx, GST and GR) and non enzymatic antioxidants (Vitamin C, Vitamin E and GSH) in the liver and kidney of control and experimental rats. The levels of enzymatic and non enzymatic antioxidant were significantly decreased in diabetic rats when compared to control rats. However, Theaflavin administration to diabetic rats significantly improved the activities of the above enzymes.

**Table 7.** Effect of theaflavin on the activities SOD, CAT, GPX and GST in control and experimental rats.

Groups	Superoxide dismutase		Catalase		Glutathione peroxidase		Glutathione-S-transferase	
	Liver	Kidney	Liver	Kidney	Liver	Kidney	Liver	Kidney
Control	7.19 ± 0.46	8.80 ± 0.50	75.24 ± 4.01	36.95 ± 3.12	8.68 ± 0.63	7.42 ± 0.58	8.53 ± 0.52	5.76 ± 0.46
Normal + Theaflavin (100 mg/kg b.wt)	7.52 ± 0.43	9.21 ± 0.51	77.00 ± 3.88	37.31 ± 3.17	9.22 ± 0.71	7.77 ± 0.57	8.92 ± 0.41	6.15 ± 0.38
Diabetes Induced	3.15 ± 0.27^b^	3.74 ± 0.35^b^	49.57 ± 3.75	24.73 ± 2.35^b^	5.19 ± 0.42	4.91 ± 0.44^b^	4.06 ± 0.31^b^	3.41 ± 0.33^b^
Diabetic + Theaflavin (100 mg/kg b.wt)	7.28 ± 0.36^c^	7.01 ± 0.40^c^	68.11 ± 4.06^c^	32.93 ± 2.81^c^	7.70 ± 0.71^c^	7.00 ± 0.53^c^	7.67 ± 0.52^c^	4.83 ± 4.63^c^
Diabetic + Metformin (500 mg/kg b.wt)	7.57 ± 0.37	7.30 ± 0.39	70.16 ± 4.26	36.33 ± 3.25	7.31 ± 0.77	7.61 ± 0.67	7.20 ± 0.46	5.21 ± 0.37

Notes: Values are given as mean ± SD for six animals in each group. Values are considered significantly different at *P* < 0.05 with post hoc LSD test *P < 0.05. The activities of enzymes are expressed as follows: SOD – One unit of activity was taken as the enzyme quantity, which gave 50% inhibition of nitroblue tetrazolium reduction in 1 min/mg protein; CAT – µmoles of H_2_O_2_ consumed/minute; GPx – µg of glutathione consumed/minute/mg protein; GST- µmoles of 1-chloro 2,4-dinitrobenzene-GSH conjugate formed/minute/mg protein.

^a^Control vs. Drug control (Theaflavin alone treated control rats).

^b^Control rats vs. Diabetic rats.

^c^Diabetic rats vs. Theaflavin treated diabetic rats.

^d^Theaflavin treated diabetic rats vs. Metformin treated diabetic rats.

**Table 8.** Effect of theaflavin on the levels of non-enzymatic antioxidants in tissues of control and experimental rats.

Groups	Vitamin C (µM/mg tissue)		Vitamin E (µM/mg tissue)		Reduced Glutathione (GSH) (µM/mg tissue)	
	Liver	Kidney	Liver	Kidney	Liver	Kidney
Control	1.35 ± 0.13	1.18 ± 0.07	0.85 ± 0.07	0.58 ± 0.04	44.12 ± 4.67	35.89 ± 3.27
Normal + Theaflavin (100 mg/kg b.wt)	1.48 ± 0.14	1.22 ± 0.09	0.95 ± 0.05	0.72 ± 0.06	45.07 ± 4.48	36.25 ± 3.34
Diabetes Induced	0.88 ± 0.067^b^	0.60 ± 0.05^b^	0.29 ± 0.02^b^	0.35 ± 0.03^b^	25.59 ± 2.15^b^	21.16 ± 1.98^b^
Diabetic + Theaflavin (100 mg/kg b.wt)	1.27 ± 0.10^c^	0.94 ± 0.04^c^	0.64 ± 0.06^c^	0.49 ± 0.04^c^	37.04 ± 3.00^c^	30.07 ± 2.57^c^
Diabetic + Metformin (500 mg/kg b.wt)	1.39 ± 0.12	1.11 ± 0.11	0.69 ± 0.05	0.57 ± 0.04	35.61 ± 3.57	32.40 ± 3.02

Notes: Values are given as mean ± SD for six animals in each group. Values are considered significantly different at *P* < 0.05 with post hoc LSD test *P < 0.05.

^a^Control vs. Drug control(Theaflavin alone treated control rats).

^b^Control rats vs. Diabetic rats.

^c^Diabetic rats vs. Theaflavin treated diabetic rats.

^d^Theaflavin treated diabetic rats vs. Metformin treated diabetic rats.

### Histopathological observations of pancreas

**Figure 3.**
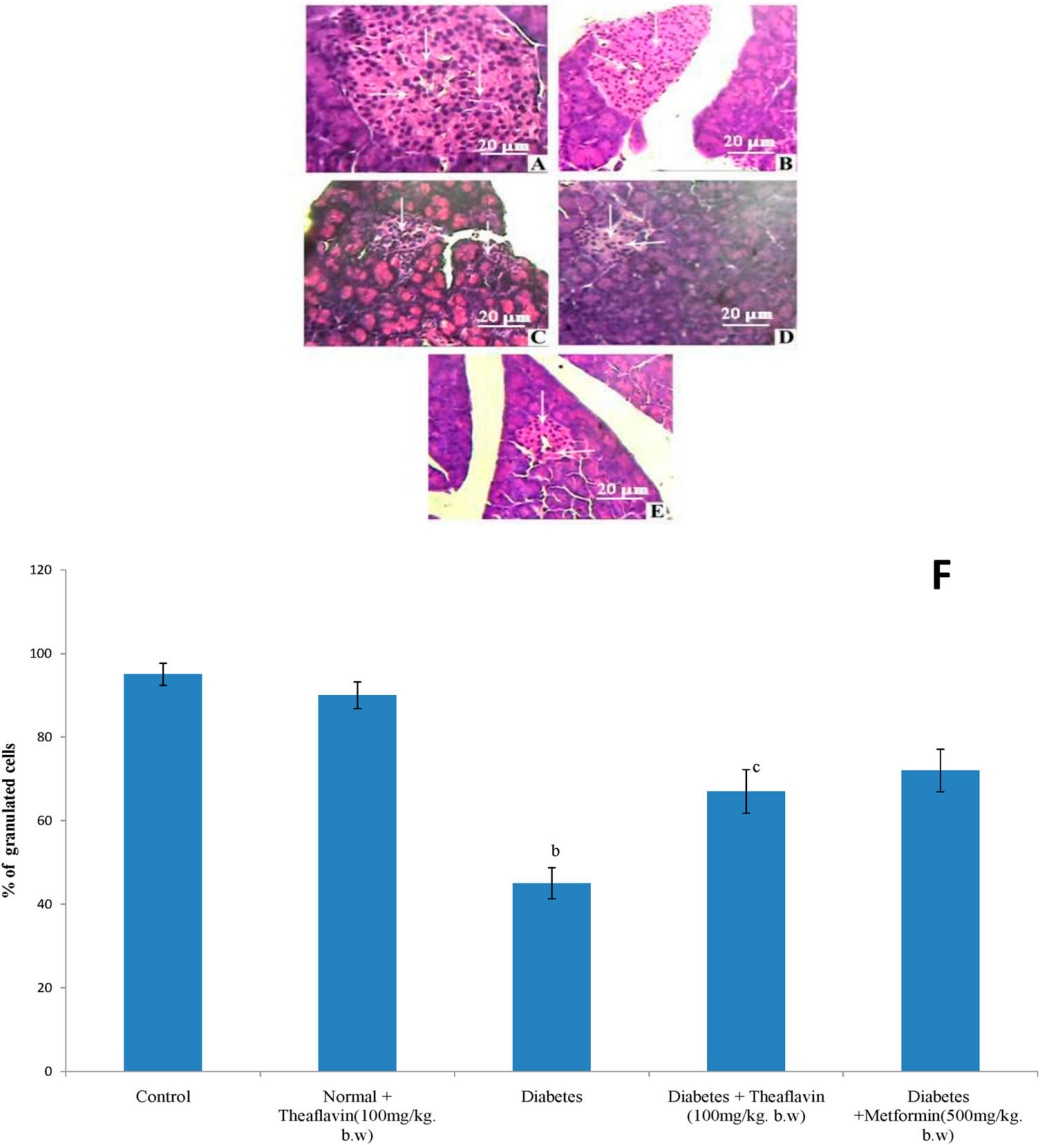
Histopathological section of pancreas of control and Experimental rats (40X). Control (A), Normal + Theaflavin (B), Diabetes induced (C) Diabetic + Theaflavin (D) Diabetic + Metformin (E). (F) Quantification of granulated cells from pancreas. Values are given as mean ± SD for six animals in each group (*n* = 6). Values are considered significantly different at *P* < 0.05 with post hoc LSD test. Comparisons are made as (a) Control vs Drug control (Theaflavin alone treated rat); (b) Control rat vs Diabetic rat; (c) Diabetic rat vs Theaflavin treated diabetic rat; (d) Theaflavin treated diabetic rat vs Metformin.

Figure 3 represents the hematoxylin and eosin staining of pancreas of control and experimental rats. Normal histological features of both exocrine and endocrine part were shown in the section of pancreatic tissue of control rats (Figure 3(A)). Pancreatic section from theaflavin alone treated rat shows no changes on beta cells and it looked like control pancreas (Figure 3(B)). The section of pancreatic tissues of diabetic rats showed degenerative changes **as well as** reduction in number and size of the islets. Subsequently, the central areas of most pancreatic islets are completely empty when compared to control rats (Figure 3(C)). The section of pancreatic tissues of diabetic rats treated with theaflavin and metformin showed marked regeneration of islets with significant number of granulated cells than that of diabetic rats. Moreover, several islets are well granulated when compared to pancreatic islets of diabetic rats indicates the improved functioning of cells which was reflected in the increased plasma insulin level (Figure 3(D) and (E)).

## Discussion

Many studies have reported that the high-fat diet fed rats develop insulin resistance and low-dose streptozotocin administration has been known to induce mild impairment of insulin secretion which mimics the natural history of type 2 diabetes. Several scientific reports have proved that the drugs can significantly reduce high blood sugar, regulate the glycogen synthesis which was very significant to maintain normal blood sugar and improve glucose tolerance. Hence, blood glucose is a key marker for diagnosis and prognosis of diabetes mellitus. Insulin insufficiency causes elevation in the levels of blood glucose as a result of excessive production of endogenous glucose by hepatic as well as extrahepatic tissues through gluconeogenic and glycogenolytic pathways and reduced consumption of glucose through glycolytic, TCA cycle, glycogenic and HMP pathways by various tissues during diabetes mellitus [41].

In the present study, upon treatment with theaflavin and metformin significantly lowered the level of blood glucose and improved the insulin levels in high fat diet and streptozotocin – induced diabetic rats. These glucose lowering and elevated insulin levels are brought by the antioxidant potential of theaflavin [7]. The consumption of natural phytochemicals containing antioxidant effect were reported to have potential health benefits and help to regenerate β cells and protect pancreatic islets against cytotoxic effects of streptozotozin [42,43].

Diabetes is characterized by loss of body weight which might be the result of augmented muscle wasting and tissue loss due to the unavailability of carbohydrate as an energy source [44]. In the present study, initial and final body weight of experimental animals were measured, whereas food and water intake were evaluated on a daily basis. Though the food and water intake of diabetic rats were increased during the experimental period, the weight gain was significantly reduced when compared to control animals. However, theaflavin and metformin administration restored the above said parameters to near normalcy. These findings clearly show that theaflavin normalizes the disturbed carbohydrate metabolism in diabetic rats by exerting its significant antidiabetic effects.

Glycosylated hemoglobin (HbA1c) is the clinical marker of chronic glycemic control in patients with diabetes mellitus [45]. Persistent hyperglycemia leads to the glycosylation of amino groups of lysine residue in proteins [46]. This condition favors reduction in the level of total hemoglobin and elevation in glycosylated hemoglobin which in turn directly proportional to blood glucose [47]. Diabetic rats showed higher levels of glycosylated hemoglobin indicating their poor glycemic control. Treatment of theaflavin and metformin to diabetic rats significantly reduced the HbA1c levels compared to untreated diabetic rats. This reflects the antioxidant potential of theaflavin and metformin in long-term control of hyperglycemia through insulin secretion.

Glycogen is the primary intracellular storable form of glucose and its level in various tissues especially in liver and skeletal muscles indicates direct reflection of insulin activity since insulin regulates glycogen storage by stimulating glycogen synthase and inhibiting glycogen phosphorylase [48]. Because streptozotocin causes destruction of β cells of islets of Langerhans resulting in marked decrease in insulin levels, the glycogen content in liver and muscle tissues decrease as the invasion of glucose in the liver is inhibited in the absence of insulin [49,50]. Our results showed that supplementation of theaflavin and metformin to diabetic rats significantly increased glycogen content of both hepatic and skeletal muscles by stimulating glycogen synthase and inhibiting glycogen phosphorylase which could be due to increased insulin levels.

Liver plays a central role in the maintenance of glucose homeostasis [51]. The uncontrolled hepatic glycogenolysis and gluconeogenesis and decreased utilization of glucose by the insulin dependent tissues are the fundamental factors contributing to a condition termed as hyperglycemia in diabetes mellitus [52]. Hyperglycemic status occurs due to the lack of suppression of hepatic glucose production in the absorptive state and excessive glucose production in the post absorptive state. The enzymes that are involved in the regulation of hepatic glucose production are potential targets for controlling the glucose homeostasis in diabetes. Hence, the current study was concentrated in evaluating the activities of hepatic key enzymes of carbohydrate metabolism in streptozotocin and high fat diet -induced diabetic rats.

Hexokinase is a major regulatory enzyme involved in the oxidation of glucose. Since it is an insulin-dependent enzyme, the hepatic hexokinase activity in diabetic rats is almost entirely inhibited or inactivated due to the absence of insulin [53]. This impairment results in a marked decline in the rate of glucose oxidation via glycolysis which ultimately leads to hyperglycemia. The markedly decreased level of insulin observed in the diabetic animals ultimately leads to the impairment in the activity of hexokinase, since insulin deficiency is a clinical imprint of diabetes [54]. Upon treatment with theaflavin and metformin to high fat diet and streptozotocin – induced rats resulted in a notable reversal in the activity of hexokinase. The diminished fasting blood glucose levels observed in theaflavin and metformin administered diabetic rats may be due to amplified hexokinase activity in the hepatic and renal tissues thereby increasing the oxidation of glucose leading to controlled glucose homeostasis.

Pyruvate kinase is a ubiquitously expressed key glycolytic enzyme that catalyzes the conversion of phosphoenolpyruvate to pyruvate with the generation of ATP and the altered expression could be expected to impair the glucose metabolism and energy production. Pyruvate kinase is regulated by its own substrate phosphoenolpyruvate and fructose-1, 6-bisphosphate, an intermediate in glycolysis which both up-regulate the Pyruvate kinase. The observed decrease in the activity of Pyruvate kinase in the liver and kidney of streptozotocin – induced diabetic rats readily accounts for the decreased utilization of glucose (glycolysis) and increased production of glucose (gluconeogenesis) by liver and kidney indicating that these two pathways are altered in diabetes [55]. Supplementation of theaflavin and metformin to high fat diet and streptozotocin -induced diabetic rats resulted in a significant increase in the activity of Pyruvate kinase. The improved activities of hexokinase and Pyruvate advocate the active utilization of glucose.

Lactate dehydrogenase is a terminal glycolytic enzyme that plays an indispensable role in the interconversion of pyruvate to lactate to yield energy under anaerobic conditions and the reaction occurs in both cytosolic and mitochondrial compartments [56,57]. Studies have shown that during diabetes, the activity of Lactate dehydrogenase increase due to impairment in insulin secretion [58]. Our results are agreement with these studies. Thus, increased activity of LDH interferes with normal glucose metabolism and insulin secretion in the β cells of pancreas and it may therefore be directly responsible for insulin secretory defects in diabetes due to the high fat diet and streptozotocin induced oxidative stress mediated cellular damage. Besides, the elevated levels of LDH in hepatic and renal tissue indicate the cell damage. Upon treatment with theaflavin and metformin to diabetic rats restored the LDH activity by regulating the NADP/NADH ratio thereby stimulating the oxidation of NADH. Normal LDH activity is indicative of improved channeling of (pyruvate) glucose for mitochondrial oxidation.

Glucose-6-phosphatase and fructose-1, 6-bisphosphatase are gluconeogenic enzymes which are involved in the homeostatic regulation of blood glucose concentration mainly in the liver and kidney and critical in providing glucose to other organs during diabetes, prolonged fasting or starvation [57]. Glucose-6-phosphatase catalyzes the dephosphorylation of glucose-6-phosphate to glucose [59]. Fructose-1, 6-bisphosphatase that catalyzes the dephosphorylation of fructose-1, 6-bisphosphate to fructose-6-phosphate serves as a site for the regulation of gluconeogenesis [60]. The increased activities of glucose-6-phosphatase and fructose-1, 6-diphosphatase in liver and kidney **of high fat diet and streptozotocin induced** diabetic rats may be due to insulin inadequacy. Theaflavin and metformin supplementation to diabetic rats significantly dwindled the activities of glucose-6-phosphatase and fructose-1, 6-diphosphatase. This might be due to improved insulin secretion which is responsible for the repression of the gluconeogenic key enzymes.

The activity of the enzyme glucose-6-phosphate dehydrogenase is usually found to be decreased in diabetic conditions which in turn slow down the pentose phosphate pathway [61]. This is consistent with our study. Administration of theaflavin and metformin to diabetic rats significantly increased the activity of glucose-6-phosphate dehydrogenase. It provides hydrogen which binds NADP^+^ and produces NADPH and enhances the synthesis of fats from carbohydrates, i.e. lipogenesis and finally the plasma glucose levels were decreased [62]. Further, the present study suggests that decrease in the activity of glucose-6-phosphate dehydrogenase is of clinical significance in the pathogenesis of diabetic complications. Treatment with theaflavin and metformin restored the activity of glucose-6-phosphate dehydrogenase to near normalcy.

Hyperglycemia results in the generation of free radicals which can exhaust antioxidant defenses thus leading to the disruption of cellular functions, oxidative damage to membranes and enhanced susceptibility to lipid peroxidation [63]. Elevated lipid peroxidation is responsible for the formation of lipid hydroperoxides. The levels of TBARS and hydroperoxides are found to be higher in diabetic individuals when compared to healthy individuals [64]. In our study, the levels of lipid peroxides and hydroperoxides were significantly increased whereas the levels of enzymatic antioxidant (SOD, CAT, GPx, GST and GR) and non enzymatic antioxidants (Vitamin C, Vitamin E and GSH) were significantly decreased in the hepatic and renal tissues of diabetic rats [65]. Oral administration of theaflavin extract significantly improved the antioxidant status by reducing the lipid peroxidation and hydroperoxides in liver and kidney tissues of diabetic rats through its antioxidative and antiperoxidative properties. Previous studies have shown that oral administration of black tea extract increased the antioxidant enzymes in pancreas, liver, kidney and cardiac tissues of diabetic rats [66]. Moreover, Leung et al. [67] have reported that theaflavin in black tea possessed the same antioxidant activity as EGCG. Theaflavin is one of the major chemical constituents of black tea and it has been proved its antioxidant properties in our studies.

## Conclusion

The results of the present study clearly indicate that oral treatment of theaflavin and metformin to diabetic rats increased the activities of hexokinase, pyruvate kinase, and glucose-6-phosphate dehydrogenase suggesting the effective utilization of glucose. The enhanced activity of glycogen synthase indicates the improved glycogen content in the liver and muscle. The reduction in the activities of gluconeogenic and glycogenolytic enzymes in hepatic and renal tissues of diabetic rats treated with the drug suggest that the drug has ameliorated the alterations in carbohydrate metabolism by preventing the endogenous glucose production through these two pathways. In addition, theaflavin protects pancreas from peroxidative injury through its antioxidant properties. The improved histological changes in the pancreas also support the parameters investigated. These findings suggest that theaflavin has complete potency to develop an antihyperglycemic agent for the treatment of diabetes mellitus. Further studies are in progress to elicit the exact mechanism behind the antihyperglycemic action of theaflavin in diabetes.
